# An initial accuracy focus reduces the effect of prior exposure on perceived accuracy of news headlines

**DOI:** 10.1186/s41235-020-00257-y

**Published:** 2020-11-05

**Authors:** Dustin P. Calvillo, Thomas J. Smelter

**Affiliations:** Psychology Department, California State University San Marcos, 333 South Twin Oaks Valley Road, San Marcos, CA 92096 USA

**Keywords:** Illusory truth effect, Fake news, Political bias, Cognitive reflection

## Abstract

The illusory truth effect occurs when the repetition of a claim increases its perceived truth. Previous studies have demonstrated the illusory truth effect with true and false news headlines. The present study examined the effects that different ratings made during initial exposure have on the illusory truth effect with news headlines. In two experiments, participants (total *N* = 575) rated a set of news headlines in one of two conditions. Some participants rated how interesting they were, and others rated how truthful they were. Participants later rated the perceived accuracy of a larger set of headlines that included previously rated and new headlines. In both experiments, prior exposure increased perceived accuracy for participants who made initial interest ratings, but not for participants who made initial truthfulness ratings. The increase in perceived accuracy that accompanies repeated exposure was attenuated when participants considered the accuracy of the headlines at initial exposure. Experiment 2 also found evidence for a political bias: participants rated politically concordant headlines as more accurate than politically discordant headlines. The magnitude of this bias was related to performance on a cognitive reflection test; more analytic participants demonstrated greater political bias. These results highlight challenges that fake news presents and suggest that initially encoding headlines’ perceived truth can serve to combat the illusion that a familiar headline is a truthful one.

## Statement of significance

One problem posed by fake news is that it misleads people, causing them to believe false information. Decades of research have shown that repeating information to people increases their perceived accuracy for that information. These findings have recently been extended to included news headlines. If people see news headlines repeatedly, they rate them as more accurate than they do headlines they have seen only once. This occurs for real and fake news headlines. The present study examined whether the type of ratings made during initial exposure can augment the propensity to evaluate previously viewed stimuli as truthful. The stimuli for the present study were news headlines and participants rated these under two different paradigms. Some participants rated how interesting the headlines were and other participants rated how truthful they were. All participants later rated the accuracy of a larger set of headlines that included previously rated and new headlines. In both experiments, headlines that were initially rated were judged as more accurate than new headlines for participants who made interest ratings, but not for participants who made truthfulness ratings. There was also evidence for a political bias. Participants rated politically concordant headlines as more accurate than political discordant headlines, and this bias increased with cognitive reflection. These results highlight challenges that fake news presents and suggest possible interventions for these challenges. Specifically, people should consider the truthfulness of news headlines they see to avoid the increase in perceived accuracy that would otherwise occur. Finally, people should be instructed about partisan biases in evaluating news headlines in an attempt to reduce these biases.

“Repetition does not transform a lie into truth”—U.S. President Franklin Delano Roosevelt

Repeating false information does not make it true. Decades of research, however, have demonstrated that repeating false claims increases their perceived accuracy. Hasher et al. (1977) first reported that repeating both true and false general knowledge statements increased their perceived accuracy. This finding has since been termed the *illusory truth effect* (also referred to as the *repetition-induced truth effect*, *truth effect*, *effect of prior exposure*, and *effect of repeated exposure*). Most explanations for the illusory truth effect claim that repeating a statement increases the fluency with which it is processed, and this processing fluency is then misattributed to the feeling of truth for the statement (e.g., Reber and Unkelbach 2010). Indeed, repetition activates the perirhinal cortex, a region of the brain associated with fluency (Wang et al. 2016). Other explanations for illusory truth posit that frequency of occurrence may be a cue to the validity of information, recognizing information and feelings of familiarity may increase belief in the information, or that repeated information creates a set of coherent references in memory, which in turn leads to greater perceived accuracy (Unkelbach et al. 2019). A meta-analysis has demonstrated the robustness of this effect (Dechêne et al. 2010). In addition, warnings sometimes reduce but do not eliminate the illusory truth effect (Nadarevic and Aßflag 2017), and knowledge of statements’ veracity does not eliminate the effect (Fazio et al. 2015). The effect has also been replicated in the context of subjective sociopolitical statements (Arkes et al. 1989) and consumer opinions (Johar and Roggeveen 2007), and it can be detected weeks (Bacon 1979; Garcia-Marques et al. 2015) and even months later (Schwartz 1982). Furthermore, the illusory truth effect occurs for implausible statements (Fazio et al. 2019), and individual differences in cognitive ability and cognitive style do not moderate this effect (De keersmaecker et al. 2020). Thus, the illusory truth effect appears to be robust across individuals, material domains, and time.

The illusory truth effect has important implications. It highlights the difficulty in understanding truth in a modern world that is rich with information that varies in its depiction and representation of the truth. With the existence of 24-h news channels, for example, false news stories may be repeated several times in a short span, which, according to the illusory truth effect, should increase their perceived accuracy among viewers. Similarly, on social media, politicians may repeat false assertions, which should increase their perceived accuracy among their followers. Fake news stories also spread rapidly on social media (Vosoughi et al. 2018), highlighting the crucial need for interventions to combat such proliferation of misinformation.

Two recent studies examined the effects of repeated exposure of news headlines on their perceived accuracy. Pennycook et al. (2018) had participants rate their willingness to share 12 news headlines (6 true and 6 false) on social media, complete filler tasks, and then rate the familiarity and accuracy of 24 news headlines (12 that had been previously rated and 12 new headlines). Pennycook et al. (2018) found that perceived accuracy was greater for those headlines previously rated than for new headlines, and this effect was similar for true and false headlines. Smelter and Calvillo (2020) had participants rate the humor of 24 headlines (12 true and 12 false), complete filler tasks, and then rate the accuracy of 48 headlines (24 old and 24 new). They replicated the effect of prior exposure on perceived accuracy from Pennycook et al. (2018). These studies demonstrated that the illusory truth effect occurs for news headlines, which was explained by repeated exposure increasing processing fluency. An important implication of these studies is that spreading fake news on social media increases its perceived accuracy.

The primary goal of the present study was to examine whether the type of ratings made during initial exposure affects the magnitude of the repeated exposure effect. Pennycook et al. (2018) and Smelter and Calvillo (2020) had different initial ratings and found different sized effects of prior exposure. Specifically, the effect of repeated exposure was larger with Smelter and Calvillo’s (2020) humor ratings that it was with Pennycook et al.’s (2018) willingness to share ratings. The particular initial ratings that participants make appear to influence the magnitude of the illusory truth effect with headlines. We speculate that when participants judge their willingness to share a headline, they may rely on some of the same cues that they would to judge truthfulness, whereas for other unrelated judgments like humor, they may utilize different cues. Willingness to share a headline is also related to its perceived accuracy (Altay et al. 2020).

In a recent study, Brashier et al. (2020) included two different initial rating tasks. They had some participants rate how interesting a set of statements were, whereas other participants rated how truthful they were. Participants then saw a larger set of statements that included the original statements and a new set of statements, and they rated their perceived accuracy for this set. Brashier et al. (2020) found the typical illusory truth effect when the initial ratings were for interest, but this effect did not occur when the initial ratings were about truthfulness. Their later experiments showed that initial truthfulness ratings only eliminated the illusory truth effect when participants had knowledge of the truth of the statements during the initial rating. Brashier et al. (2020) concluded that the illusory truth effect disappears when participants think about statements’ truth at initial exposure, particularly when they have the knowledge to recognize that false statements are not true. The increase in fluency associated with repeated exposure does not lead to increased perceived accuracy when individuals focus on the accuracy of information at initial encoding.

In the present study, we extended Brashier et al.’s (2020) method to news headlines. We examined whether asking participants to consider the truthfulness of news headlines at initial exposure reduces the effects of prior exposure on perceived accuracy reported by Pennycook et al. (2018) and Smelter and Calvillo (2020). If our prediction is supported, this finding would suggest that a strategy to reduce the impact of the spread of fake news on social media would be to encourage people to think about the truthfulness of news stories that they see. These findings can also inform theoretical explanations of the illusory truth effect.

## Preregistration and ethics information

Before data collection for each experiment, we preregistered our hypotheses, data collection plans, inclusion criteria, and planned analyses on the Open Science Framework (OSF). We note our exploratory analyses that were not preregistered in the Results sections of each experiment. Furthermore, the materials and data from both experiments are available on the OSF (https://osf.io/8xvdy/). The experiments described in this manuscript were approved by an Institutional Review Board prior to data collection and all participants consented to their participation and to their de-identified data being posted on the OSF.

## Experiment 1

In Experiment 1, participants initially rated headlines in one of two conditions. Some participants rated how interesting the headlines were, whereas other participants rated how truthful they were. All participants then rated the accuracy of a larger set of headlines that included the previously rated and new headlines. The primary hypothesis was the interaction between initial rating task and initial exposure. Specifically, we predicted that when the initial rating task was interest, there would be an effect of initial exposure, such that repeated headlines would result in greater perceived accuracy than would new headlines; but when the initial rating task was truthfulness, there would not be an effect of initial exposure. In other words, the illusory truth effect would be present for participants who made initial interest ratings; but absent for participants who made initial truthfulness ratings. We also predicted that there would be a main effect of prior exposure (repeated headlines would result in greater perceived accuracy than new headlines) and a main effect of headline truth (true headlines would result in greater perceived accuracy than false headlines). We did not predict any other interactions.

### Methods

#### Power analysis

To determine our sample size, we conducted a power analysis. The two previous studies that examined the effects of prior exposure on perceived accuracy of news headlines found effect sizes of *η*_p_^2^ = .09 and *η*_p_^2^ = .20 (Pennycook et al. 2018; Smelter and Calvillo 2020, respectively). Using G*Power 3 (Faul et al. 2007), we calculated that we needed 82 participants per condition to have a power of 0.80 to detect the smaller of these two effect sizes. Thus, we aimed for total of 164 participants (with 82 in each condition). After data collection, we realized that our power analysis was based on the ability to detect an illusory truth effect in a specific group, rather than to detect an interaction between groups. Therefore, this study may have been underpowered to test our primary hypothesis.

#### Participants

We preregistered two inclusion criteria (described in “Materials and procedure” section). A total of 212 Mechanical Turk workers completed this experiment, and 172 met both inclusion criteria. Of these 172 participants, 83 identified as female and 89 identified as male. Participants ranged in age from 19 to 78, with a median of 36 years, and all participants claimed that they resided in the USA.

#### Design

The design of Experiment 1 was a 2 (initial rating task: interest, truthfulness) × 2 (prior exposure: repeated, new) × 2 (headline truth: true, false) mixed-model factorial. Initial rating task was manipulated between-subjects and initial exposure and headline were manipulated within-subjects. Eighty-five participants were in the interest rating condition and 87 were in the truthfulness rating condition.

#### Materials and procedure

**Fig. 1:**
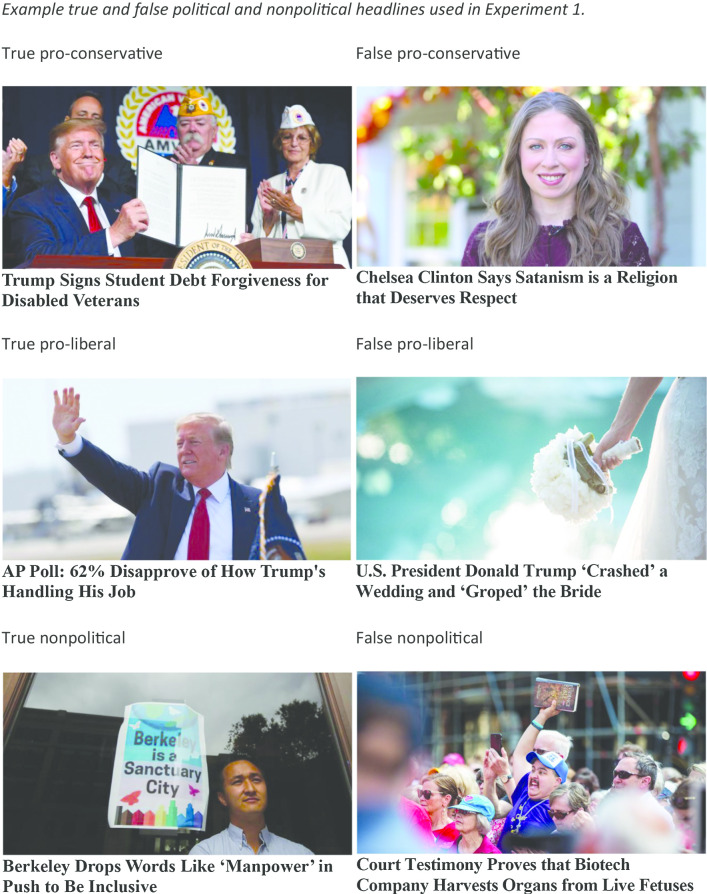
Example true and false political and nonpolitical headlines used in Experiment 1

The materials consisted of 32 news headlines, 16 true and 16 false. The true headlines were taken from the website USNews.com, whereas the false headlines were taken from the fact-checking website Snopes.com. The headlines were edited to be in the same font and all accompanying pictures were edited to be the same size. We included pictures with headlines because most fake news studies have included pictures (e.g., Pennycook et al. 2018), although the inclusion of these pictures has been shown to increase perceived accuracy of both true and false headlines (Smelter and Calvillo 2020). All false headlines had received a *false* rating from Snopes. All headlines appeared on their respective websites in July, August, and September of 2019. The headlines were a mixture of political and nonpolitical headlines, and the political headlines contained some that were pro-liberal and some that were pro-conservative. We selected false headlines with the intent of capturing a representative set of fake news that existed at the time. To select true headlines that were somewhat implausible, we used the Offbeat section of US News for many of them. We also included some true political headlines (from US News) so that there were some true and some false political headlines. Figure 1 contains examples of true and false headlines that were pro-conservative, pro-liberal, and nonpolitical. The entire set of headlines is available on the OSF page for this study. Headlines differ from typical materials used in illusory truth studies. Headlines’ truth may be easier to judge based on knowledge than typically used general knowledge statements, but previous studies have shown the illusory truth effect with headlines (Pennycook et al. 2018; Smelter and Calvillo 2020).

Participants were randomly assigned to either the interest or truthfulness rating condition. Participants then rated 16 headlines, 8 true and 8 false. We counterbalanced which 16 headlines from the larger set of 32 they rated. Participants made their initial ratings on a 6-point scale, either from *very uninteresting* to *very interesting* or from *definitely false* to *definitely true*. This was the same initial rating scale used by Brashier et al. (2020). Immediately after initial ratings, participants rated the accuracy of all 32 headlines (for 16 they had made previous ratings and the other 16 were new) on a 4-point scale from *not at all accurate* to *very accurate*. We used a different scale for final ratings for two reasons. First, this is the same scale commonly used in fake news studies (e.g., Pennycook et al. 2018) and using the same scale facilitates comparison across studies. Second, we used different initial and final rating scales to prevent participants from remembering and duplicating their initial responses. The lack of delay between initial and final ratings is similar to the procedure of Brashier et al. (2020). After completing the final accuracy ratings, participants answered some demographic questions (age, gender) and two honesty questions. Participants were asked if they had responded randomly or without reading any questions in the study and if they had looked up any headlines online. Participants who responded *yes* to either question were omitted from analysis (*n* = 40). Finally, participants were debriefed and paid for their participation. We conducted this experiment with TurkPrime (Litman et al. 2016).

### Results and discussion

We conducted a three-way mixed-model ANOVA with initial rating task, prior exposure, and headline truth as independent variables and perceived accuracy as the dependent variable. Table 1 contains the mean perceived accuracy for each condition. We found a main effect of prior exposure*, F*(1, 170) = 8.39, *p* = .004, *η*_p_^2^ = .05. Repeated headlines (*M* = 2.57, 95% CI [2.50, 2.63]) resulted in greater perceived accuracy than new headlines (*M* = 2.48, 95% CI [2.40, 2.54]). We also found a significant main effect of headline truth, *F*(1, 170) = 163.33, *p* < 0.001, *η*_p_^2^ = .49. True headlines (*M* = 2.77, 95% CI [2.71, 2.83]) resulted in greater perceived accuracy than false headlines (*M* = 2.27, 95% CI [2.19, 2.35]). The specific type of initial ratings did not significantly affect final perceived accuracy, *F*(1, 170) = 0.13, *p* = .718, *η*_p_^2^ = .00.

**Table 1:** Mean perceived accuracy based on whether headlines were true or false, whether they were new or repeated, and whether the initial ratings were about interest of truthfulness from Experiment 1

Initial rating	Headline truth	New		Repeated	
		*M*	95% CI	*M*	95% CI
Interest					
	True	2.65	[2.53, 2.76]	2.87	[2.76, 2.97]
	False	2.23	[2.11, 2.36]	2.30	[2.17, 2.42]
Truthfulness					
	True	2.76	[2.65, 2.87]	2.81	[2.71, 2.91]
	False	2.27	[2.15, 2.40]	2.29	[2.17, 2.41]

**Fig. 2:**
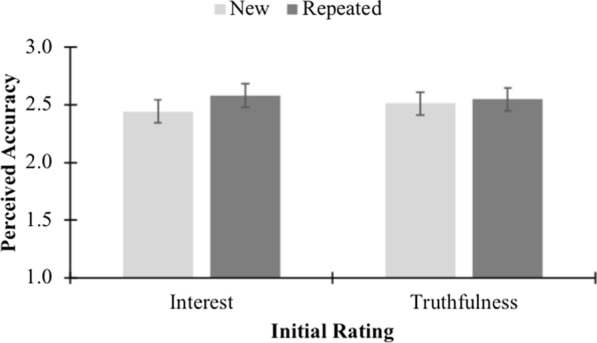
Differences between perceived accuracy of new and repeated headlines for both initial rating conditions in Experiment 1 (error bars show 95% CIs)

The primary hypothesis was that there would be an interaction between prior exposure and initial ratings. Specifically, we predicted that repeated headlines would be perceived as more accurate than new headlines when the initial rating was about interest, but not when it was about truthfulness. This interaction was not statistically significant, *F*(1, 170) = 3.00, *p* = .085, *η*_p_^2^ = .02. Because this interaction was nearly significant, we conducted simple effects tests to examine the specific simple effects that we predicted. These analyses were exploratory and not preregistered. Figure 2 displays the relevant means. For participants who made initial interest ratings, their subsequent perceived accuracy was greater for repeated headlines than for new headlines, *t*(84) = 3.01, *p* = .003, *d* = 0.33. This was not the case for those who made initial truthfulness ratings, *t*(86) = 0.91, *p* = .367, *d* = 0.10. Repeated headlines and new headlines had similar perceived accuracy. No other interactions were significant.

## Experiment 2

The primary purpose of Experiment 1 was to examine the interaction between initial ratings and prior exposure. The interaction was not significant, but simple effects tests were consistent with our hypothesis. However, Experiment 1 may have been underpowered to detect this interaction. The power analysis was based on the number of participants needed in a condition to detect an effect of prior exposure. There were no relevant data available for the interaction. The primary purpose of Experiment 2 was to again test the interaction between initial ratings and prior exposure, using the interaction effect size of Experiment 1 to sufficiently power a test of this interaction. The secondary purpose of Experiment 2 was to examine if participants’ political ideology would affect their ratings of perceived truth for news headlines. Previous investigations have found that people perceive politically concordant headlines as more accurate than politically discordant headlines (Pennycook et al. 2018; Pennycook and Rand 2019). A similar finding has been referred to as a *political bias* (Faragó et al. 2019).

In Experiment 2, we used a set of all political headlines and we included an equal number of pro-liberal and pro-conservative headlines to examine political bias. We also included a measure of cognitive reflection in Experiment 2. Fake news studies have found that cognitive reflection predicts better discernment of true and false headlines (Bronstein et al. 2019; Pennycook and Rand 2019, 2020). We attempted to replicate this finding in Experiment 2 and to examine how cognitive reflection relates to political bias. This political bias shares some similarity with other phenomena, such as *myside bias*, *belief bias*, and *motivated reasoning*. Myside bias occurs when individuals’ prior attitudes and opinions bias how they evaluate and generate evidence (Stanovich et al. 2013), belief bias occurs when participants accept more believable conclusions than unbelievable conclusions, independent of the conclusions’ validity (Evans et al. 1983), and motivated reasoning occurs when participants’ preferences affect their evaluation of evidence or decisions (Kunda 1990). In each of these, participants’ prior knowledge or attitudes bias their performance. We believe something similar occurs with political bias in headline judgments: participants perceive ideologically consistent headlines as more accurate than ideologically inconsistent headlines. According to the extant literature on each domain, cognitive ability (often measured by cognitive reflection) has distinctive relationships with these different phenomena: cognitive ability is unrelated to myside bias (e.g., Stanovich and West 2007, 2008), it is negatively related to belief bias (e.g., Toplak et al. 2011), and it is positively related to motivated reasoning (e.g., Kahan 2013); participants with greater cognitive ability engage in more motivated reasoning. Because of the similarity between political bias, myside bias, belief bias, and motivated reasoning, and the associations of the latter three with cognitive ability, we examined the relationship between cognitive reflection and political bias.

In Experiment 2, our primary prediction was the interaction between rating and prior exposure. Specifically, we predicted that with initial interest ratings, prior exposure would increase subsequent perceived accuracy, but with initial truthfulness ratings, prior exposure would not affect subsequent perceived accuracy. We also predicted a main effect of prior exposure (repeated headlines would result in greater perceived accuracy than new headlines) and of headline truth on perceived accuracy (true headlines would result in greater perceived accuracy than false headlines). We also expected to find evidence for political bias: perceived accuracy would be greater for politically concordant headlines than for politically discordant headlines. Finally, we predicted that cognitive reflection performance would predict news discernment, and we examined how cognitive reflection performance related to political bias.

### Methods

#### Power analysis

We conducted a power analysis to determine our sample size. We used the interaction effect size from Experiment 1 (*η*_p_^2^ = .02). Using G*Power 3 (Faul et al. 2007), we found that we needed 387 participants to have a power of 0.80 to detect this interaction. Thus, we aimed for total of 388 participants (with 194 in each condition).

#### Participants

We preregistered the same two inclusion criteria as in Experiment 1. A total of 413 Mechanical Turk workers completed this experiment, and 403 met both inclusion criteria. Of these 403 participants, 233 identified as female, 166 identified as male, 3 identified as another gender, and 1 declined to respond to the gender question. Additionally, 189 identified as Democrats, 113 identified as Republicans, and 101 identified as neither. Participants ranged in age from 19 to 77, with a median of 38 years, and all participants claimed that they resided in the USA.

#### Design

The design of Experiment 2 was a 2 (initial rating task: interest, truthfulness) × 2 (prior exposure: previously rated, new) × 2 (headline truth: true, false) mixed-model factorial. Initial rating task was manipulated between-subjects and initial exposure, and headline truth was manipulated within-subjects. One hundred ninety-nine participants were in the interest rating condition and 204 were in the truthfulness rating condition.

#### Materials and procedure

The materials included 32 news headlines, 16 true and 16 false. Again, the true headlines were taken from the website USNews.com, whereas the false headlines were taken from the fact-checking website Snopes.com. All false headlines had received a *false* rating from Snopes. All headlines appeared on their respective websites between November 2018 and September 2019, and all headlines were edited to have the same font and picture size. Unlike in Experiment 1, the headlines in Experiment 2 were all political and contained an equal number of pro-liberal and pro-conservative true and false headlines. The entire set of headlines is available on the OSF page for this study and in Additional file 1. Experiment 2 also included a cognitive reflection test (CRT). We selected seven CRT items that had provided good variability with Mechanical Turk workers in our previous studies that came from four sources (Baron et al. 2015; Oldrati et al. 2016; Primi et al. 2016; Thomson and Oppenheimer 2016). The specific CRT items are included on the OSF page for this study.

The procedure was similar to that of Experiment 1. Participants were randomly assigned to either the interest or truthfulness rating conditions and then rated 16 headlines (8 true and 8 false) on the same 6-point scales as Experiment 1. We counterbalanced which headlines received initial ratings. After these initial ratings, participants answered some demographic questions (age, gender, political party), some political ideology questions, and then completed the 7-item CRT. Participants then rated the accuracy of all 32 headlines (16 previously rated and 16 new) on the same 4-point scale as Experiment 1. After completing the final accuracy ratings, participants answered the same two honesty questions as those in Experiment 1. Ten participants failed at least one honesty check question. Finally, participants were debriefed and paid for their participation. We conducted this experiment with TurkPrime (Litman et al. 2016).

### Results and discussion

To test our main hypotheses, we conducted a three-way mixed-model ANOVA with initial rating task, prior exposure, and headline truth as independent variables and perceived accuracy as the dependent variable. Table 2 contains the mean perceived accuracy for each condition. We found a main effect of prior exposure*, F*(1, 401) = 31.29, *p* < 0.001, *η*_p_^2^ = .07. Repeated headlines (*M* = 2.54, 95% CI [2.50. 2.57]) resulted in greater perceived accuracy than new headlines (*M* = 2.43, 95% CI [2.39. 2.47]). We also found a significant main effect of headline truth, *F*(1, 401) = 819.99, *p* < 0.001, *η*_p_^2^ = .67. True headlines (*M* = 2.79, 95% CI [2.76, 2.83]) resulted in greater perceived accuracy than false headlines (*M* = 2.18, 95% CI [2.14, 2.21]). The specific type of initial ratings did not have a significant main effect on final perceived accuracy, *F*(1, 401) = 1.32, *p* = .251, *η*_p_^2^ = .00.

**Table 2:** Mean perceived accuracy based on whether headlines were true or false, whether they were new or repeated, and whether the initial ratings were about interest of truthfulness from Experiment 2

Initial rating	Headline truth	New		Repeated	
		*M*	95% CI	*M*	95% CI
Interest					
	True	2.75	[2.69, 2.82]	2.90	[2.84, 2.96]
	False	2.07	[2.01, 2.14]	2.28	[2.21, 2.34]
Truthfulness					
	True	2.72	[2.66, 2.79]	2.79	[2.73, 2.85]
	False	2.17	[2.11, 2.23]	2.18	[2.12, 2.24]

**Fig. 3:**
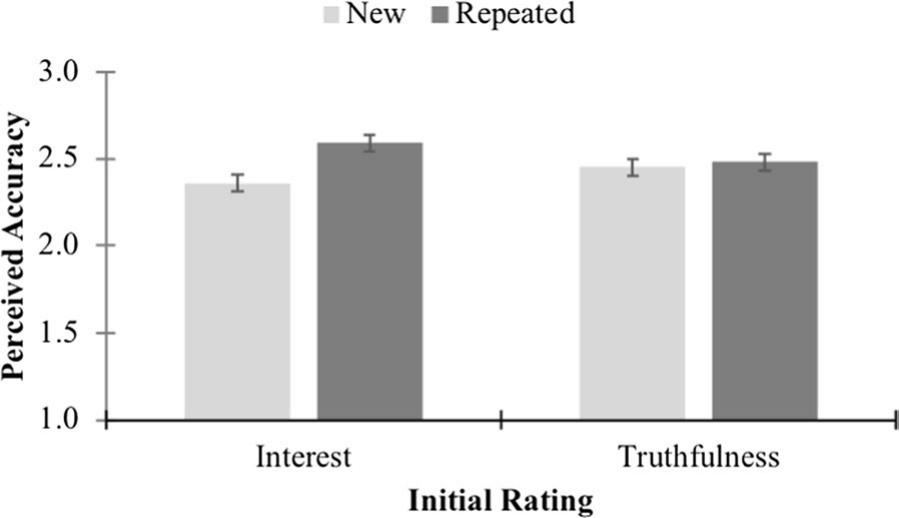
Differences between perceived accuracy of new and repeated headlines for both initial rating conditions in Experiment 2 (error bars show 95% CIs)

Our primary hypothesis was that there would be an interaction between prior exposure and initial rating. Specifically, we predicted that repeated headlines would be rated as more accurate than new headlines when the initial rating was about interest, but not when it was about truthfulness. This interaction was significant, *F*(1, 401) = 12.98, *p* < 0.001, *η*_p_^2^ = .03. Figure 2 displays the relevant means. We conducted simple effects tests to examine the specific simple effects that we predicted. As predicted, for participants who made initial interest ratings, subsequent perceived accuracy was greater for repeated headlines than for new headlines, *t*(198) = 5.36, *p* < .001, *d* = 0.38. This was not the case for those who made initial truthfulness ratings, *t*(203) = 1.90, *p* = .059, *d* = 0.13. For those participants, repeated headlines and new headlines resulted in similar perceived accuracy (Fig. 3).

The two-way interactions between initial rating and headline truth and between prior exposure and headline truth were not significant; *F*(1, 401) = 3.05, *p* = .081, *η*_p_^2^ = .01; *F*(1, 401) = 0.02, *p* = .885, *η*_p_^2^ = .00, respectively. The three-way interaction between initial rating, prior exposure, and headline truth was significant, *F*(1, 401) = 3.96, *p* = .047, *η*_p_^2^ = .01. To explore this interaction, we examined the effects of initial rating and prior exposure separately for true and false headlines. This interaction was unexpected, and these simple effects tests were not preregistered. With true headlines, the two-way interaction between initial rating and prior exposure was not significant, *F*(1, 401) = 2.88, *p* = .091, *η*_p_^2^ = .01, whereas this interaction was significant with false headlines, *F*(1, 401) = 18.22, *p* < 0.001, *η*_p_^2^ = .04.

We also examined how the concordance of the headlines to participants’ ideology affected accuracy ratings by adding whether headlines were concordant or discordant. For participants who identified as Republicans, we coded pro-conservative headlines as concordant and pro-liberal headlines as discordant, and we did the opposite for participants who identified as Democrats. The participants who identified as neither Republican nor Democrat are excluded from these analyses. These analyses deviated from our preregistered plan. The factor of headline concordance was added to the ANOVA that we analyzed in the previous paragraphs in a four-way ANOVA. Participants demonstrated a political bias. They perceived politically concordant headlines (*M* = 2.77, 95% CI [2.72, 2.83]) as more accurate than discordant headlines (*M* = 2.21, 95% CI [2.17, 2.27]), *F*(1, 300) = 260.16, *p* < 0.001, *η*_p_^2^ = .46. Concordance interacted with headline truth, *F*(1, 300) = 15.53, *p* < 0.001, *η*_p_^2^ =.05. This interaction appears to have occurred because the effect of political concordance was greater with false headlines (concordant *M* = 2.50, 95% CI [2.44, 2.56]; discordant: *M* = 1.88, 95% CI [1.82, 1.94]) than it was with true headlines (concordant *M* = 3.05, 95% CI [2.99, 3.11]; discordant: *M* = 2.56, 95% CI [2.51, 2.62]). Concordance did not interact with any other variables in two-way interactions, three-way interactions, or the four-way interaction.

We then examined CRT performance. Overall, participants correctly answered a mean of 3.35 (out of 7) CRT items correctly (Cronbach’s *α* = 0.61; participants responded with the intuitive answer to 2.17 items, on average). CRT performance was positively correlated with news discernment, *r*(401) = .26, *p* < 0.001. Next, we explored how CRT performance related to political bias. We preregistered this analysis as exploratory. To calculate political bias, we subtracted the perceived accuracy of politically discordant headlines from that of concordant headlines. CRT performance was positively correlated with political bias, *r*(300) = .12, *p* = .036. Thus, more analytic participants showed greater political bias. To further explore this relationship, we examined the relationship between CRT performance and true and false news that was politically concordant and discordant. CRT performance was positively correlated with ratings of politically concordant true news, *r*(300) = .16, *p* = .005, negatively correlated with ratings of politically discordant fake news, *r*(300) = − 0.20, *p* < 0.001, and not significantly correlated with politically concordant fake news, *r*(300) = − 0.07, *p* = .252, or with politically discordant true news, *r*(300) = .01, *p* = .864. These results show that more analytic participants show greater political bias because they are more likely to perceive concordant true news as accurate and less likely to perceive discordant fake news as accurate. In Additional file 1, we also report mean perceived accuracy for each headline based on whether participants were Democrats, Republicans, or neither, and the correlations between CRT performance and perceived accuracy for each group. These analyses were not preregistered.

## General discussion

The present study examined the illusory truth effect with news headlines. Replicating previous studies (Pennycook et al. 2018; Smelter and Calvillo 2020), we found that prior exposure to fake news increased perceived accuracy. Our primary goal was to extend the findings of Brashier et al. (2020) to evaluations of news headlines. Brashier et al. (2020) found that repeated exposure had the typical effect when initial ratings were about participants’ interest, but this effect was diminished when the initial ratings were about truthfulness. We found the same pattern in the present study. The predicted interaction failed to reach significance in Experiment 1, which was likely underpowered, but the simple effects tests were consistent with predictions. We increased power in Experiment 2 and found the same pattern and a significant interaction between prior exposure and initial rating. These results are consistent with meta-analytic findings that the illusory truth effect is smaller in studies that included initial ratings of truthfulness (Dechêne et al. 2010). It is important to note that the effects of repeated exposure on perceived accuracy were in the small to medium range (*d* = 0.33 and *d* = 0.38 in Experiments 1 and 2, respectively; Cohen 1992) for participants who made initial interest ratings. These effects were similar in size to those previously reported (Pennycook et al. 2018; Smelter and Calvillo 2020), and suggest that repeated exposure to headlines modestly increases perceived accuracy.

Our secondary goal was to examine political bias in judgments of headlines’ accuracy. In Experiment 2, we found evidence for political bias in headline evaluations. Politically concordant headlines resulted in greater perceived accuracy than political discordant headlines. These results replicate those from previous studies (Pennycook et al. 2018; Pennycook and Rand 2019). We also replicated previous reports that CRT performance predicted news discernment (Bronstein et al. 2019; Pennycook and Rand 2019, 2020). Although we did not predict it, we found that CRT performance was positively correlated with political bias. More analytic participants demonstrated greater political bias. This relationship resulted from more analytic participants perceiving political concordant true headlines as more accurate and politically discordant headlines as less accurate. Interestingly, greater cognitive reflection performance is related to both better news discernment and judgments that are more biased towards’ participants political ideology. More research is needed to better understand the relationship between CRT performance and political bias.

Brashier and Marsh (2020) reviewed the literature on how people judge truth. They identified three cues that influence truth judgments: base rates, memories, and feelings. These cues can explain how participants judge the accuracy of news headlines. Because most headlines that people have encountered have been true, the base rate truth of headlines should bias participants to believe that a headline is true. Cues from memories and feelings can then update beliefs about the truth of a headline. Therefore, according to this conceptualization, participants ought to believe that headlines are accurate if they match the content in their memory, and they should disbelieve headlines that contradict their memory contents. For example, if participants have read news stories from trusted sources, they may be more likely to believe subsequently related headlines that they encounter. Finally, participants take cues from the feelings elicited by the headlines. The effects reported in the present study concern participants’ feelings. Repeated exposure increases processing fluency, and the feeling of fluency serves as a cue for truth. Initial truth ratings, however, can allow participants to discount fluency. We believe that political bias also arises from participants’ feelings. Specifically, participants experience more positive feelings with politically concordant and more negative feelings with politically discordant headlines. Collectively then, based on the model described by Brashier and Marsh (2020), we believe that participants start with a base rate biased toward rating headlines as accurate and then update them based on a memory search for relevant information and the feelings that accompany the headlines.

The results of the present study have important implications for the effects of exposure to fake news. In order to reduce the illusory truth effect that occurs when news headlines are repeatedly observed, people should think about the truth of each headline they encounter. Even though the interestingness of online information is something that affects its likelihood of being shared, as evidenced by the fact that content is more likely to spread on Twitter if it is viewed as interesting (Bakshy et al. 2011), the present study nonetheless demonstrates that there are substantial risks of evaluating news and internet content in such a way. Additionally, people should consider their ideological biases. Reducing bias among ideologues may reduce extremism, and reducing extremism has been identified as one of psychological science’s most imperative goals (Lilienfeld et al. 2009). Confirmation bias can be reduced with brief interventions, and this reduction can last at least 2 months (Morewedge et al. 2015). Future research should address the efficacy of debiasing political bias in the context of news accuracy judgments.

It is encouraging that individuals’ perceived accuracy was greater for true headlines than it was for false headlines, replicating previous studies (e.g., Pennycook and Rand 2019; Smelter and Calvillo 2020). Nonetheless, it does seem that people struggle to stay vigilant when consuming information (e.g., Pennycook and Rand 2019) and that, through this laziness, individuals may leave themselves vulnerable to be influenced by factors other than accuracy—like interestingness. For example, Pennycook et al. (2019) suggested that people often do not intentionally spread misinformation, but instead may be influenced by factors other than truthfulness when deciding what to share. Recent investigations speak to this suggestion by showing that asking people to consider the accuracy of information reduces the sharing of fake news (Fazio 2020; Pennycook et al. 2019, 2020). Thus, our investigation presents another simple and scalable benefit of prompting the critical evaluation of news content.

## Conclusion

In two preregistered studies, we found that the effects of prior exposure to news headlines on perceived accuracy can be reduced if participants consider headlines’ truth at the initial exposure. We also found a political bias in news headline evaluations, such that participants rated politically concordant headlines as more accurate than political discordant headlines. This political bias was larger among participants with greater cognitive reflection. These findings highlight some of the challenges for combating the effects of fake news and suggest some possible interventions for these effects. Further, the need for effective interventions is pressing given that fake news has been shown to influence individuals’ attitudes toward real-world issues, including candidates for political office (Bovet and Makse 2019), public policy issues (Bastos and Mercea 2019), and health-related information (Iacobucci 2019). The findings of the present study suggest that assessing news headlines’ interest can increase susceptibility of false information. The task of wading through an increasingly information-dense world may often feel daunting, especially given the pervading influence of the illusory truth effect and the abundance of misinformation. However, we show that initially evaluating news headlines for accuracy can help to combat the illusion that a familiar headline is a truthful one.

## Supplementary information

**Additional file 1.** Exploratory analyses for Experiment 2.

## Data Availability

The materials used and datasets generated during the current study are available on the Open Science Framework at https://osf.io/8xvdy/.
