# Supplementary material for: An initial accuracy focus reduces the effect of prior exposure on perceived accuracy of news headlines
Source: Cogn Res Princ Implic. 2020 Nov 5;5:55. doi: 10.1186/s41235-020-00257-y (PMC7644737; doi:10.1186/s41235-020-00257-y)
Supplement: Supplementary file 1 — Additional file 1. Exploratory analyses for Experiment 2. [file 41235_2020_257_MOESM1_ESM.docx]

**Supplementary Analyses: Experiment 2 Ratings by Political Party and Correlations with CRT**

**Headline 01: True, pro-liberal Headline 02: True, pro-liberal**


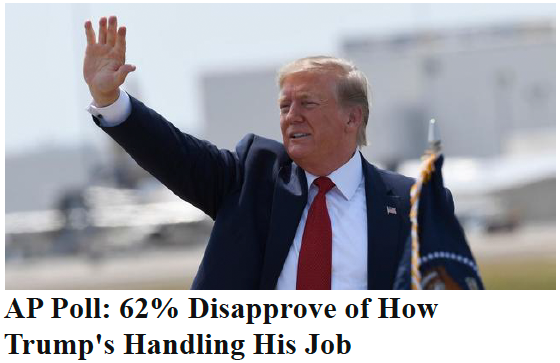

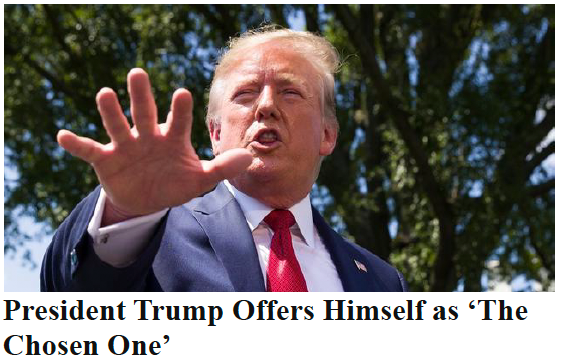


Democrat: *M* = 3.39, *SD* = 0.74, *r*(188) = .16, *p* = .033 Democrat: *M* = 2.94, *SD* = 1.09, *r*(188) = .02, *p* = .757

Republican: *M* = 2.49, *SD* = 1.13, *r*(111) = -.06, *p* = .509 Republican: *M* = 2.11, *SD* = 1.13, *r*(110) = -.01, *p* = .938

Neither: *M* = 2.96, *SD* = 0.86, *r*(98) = .24, *p* = .015 Neither: *M* = 2.52, *SD* = 1.08, *r*(98) = -.14, *p* = .176

**Headline 03: True, pro-liberal Headline 04: True, pro-liberal**

**
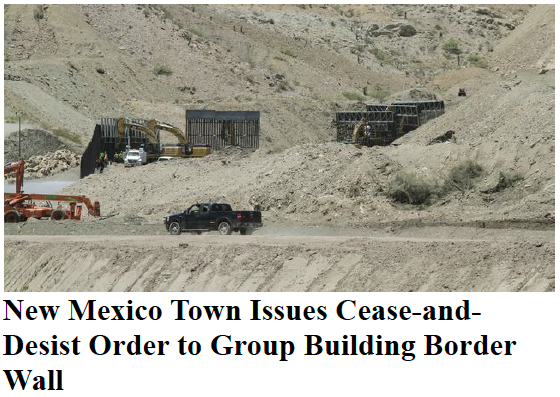

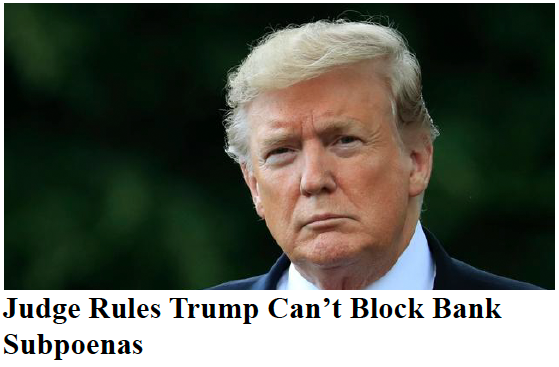
**

Democrat: *M* = 2.81, *SD* = 0.88, *r*(188) = .10, *p* = .166 Democrat: *M* = 3.08, *SD* = 0.85, *r*(187) = .24, *p* = .001

Republican: *M* = 2.74, *SD* = 1.13, *r*(111) = .07, *p* = .443 Republican: *M* = 2.71, *SD* = 0.91, *r*(110) = -.03, *p* = .726

Neither: *M* = 2.54, *SD* = 0.74, *r*(98) = .03, *p* = .789 Neither: *M* = 2.81, *SD* = 0.71, *r*(98) = .04, *p* = .702

**Headline 05: True, pro-conservative Headline 06: True, pro-conservative**

**
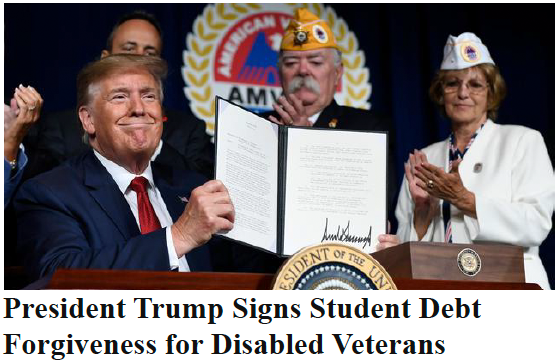

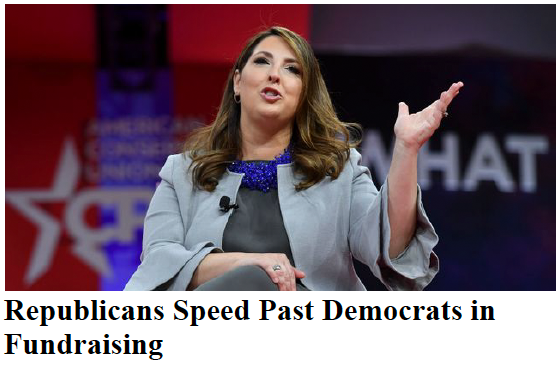
**

Democrat: *M* = 2.28, *SD* =1.00, *r*(188) = .-.11, *p* = .127 Democrat: *M* = 2.67, *SD* = 0.82, *r*(188) = .05, *p* = .498

Republican: *M* = 3.04, *SD* = 0.95, *r*(111) = .12, *p* = .221 Republican: *M* = 3.04, *SD* = .87, *r*(111) = .06, *p* = .505

Other: *M* = 2.70, *SD* = 0.94, *r*(97) = .01, *p* = .928 Other: *M* = 2.73, *SD* = 0.78, *r*(98) = .11, *p* = .298

**Headline 07: True, pro-conservative Headline 08: True, pro-conservative**

**
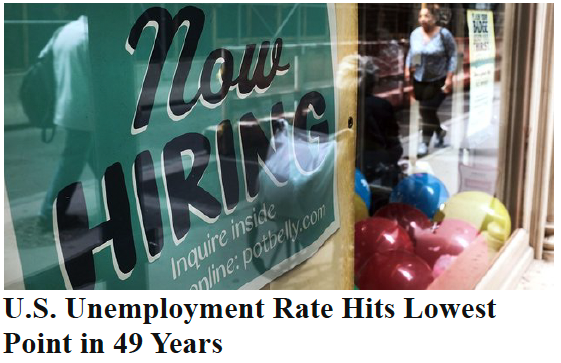

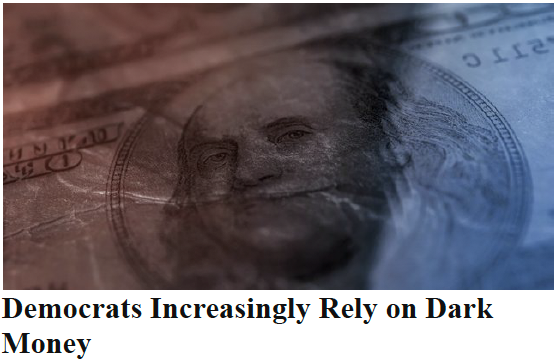
**

Democrat: *M* = 2.73, *SD* = 0.94, *r*(188) = .14, *p* = .056 Democrat: *M* = 1.80, *SD* = 0.86, *r*(188) = .00, *p* = .964

Republican: *M* = 3.25, *SD* = 0.98, *r*(111) = .07, *p* = .494 Republican: *M* = 2.54, *SD* = 0.98, *r*(111) = -.09, *p* = .348

Other: *M* = 3.04, *SD* = 0.84, *r*(98) = .05, *p* = .595 Other: *M* = 2.14, *SD* = 0.80, *r*(98) = 09, *p* = .396

**Headline 09: False, pro-liberal Headline 10: False, pro-liberal**

**
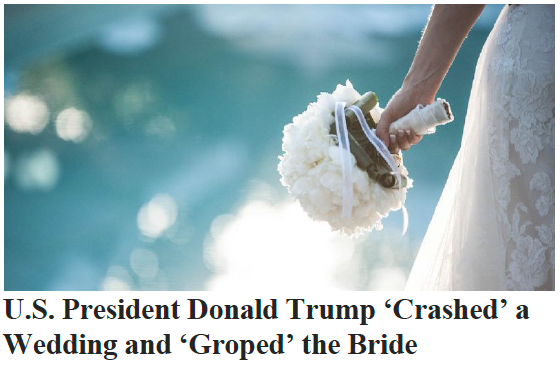

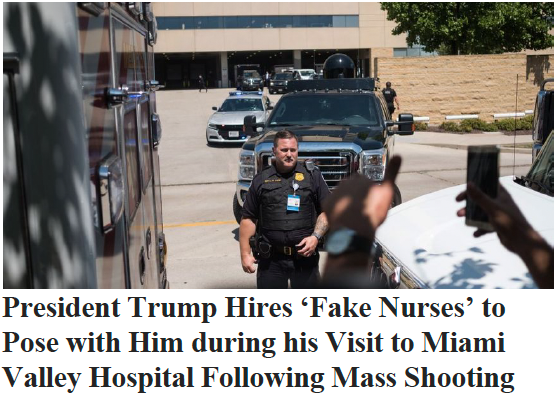
**

Democrat: *M* = 2.04, *SD* = 1.03, *r*(188) = .03, *p* = .715 Democrat: *M* = 2.15, *SD* = 0.94, *r*(187) = .07, *p* = .357

Republican: *M* = 1.41, *SD* = 0.70, *r*(111) = -.03, *p* = .793 Republican: *M* = 1.78, *SD* = 0.89, *r*(111) = -.14, *p* = .138

Other: *M* = 1.54, *SD* = 0.75, *r*(97) = -.14, *p* = .157 Other: *M* = 1.80, *SD* = 0.78, *r*(98) = -.05, *p* = .642

**Headline 11: False, pro-liberal Headline 12: False, pro-liberal**

**
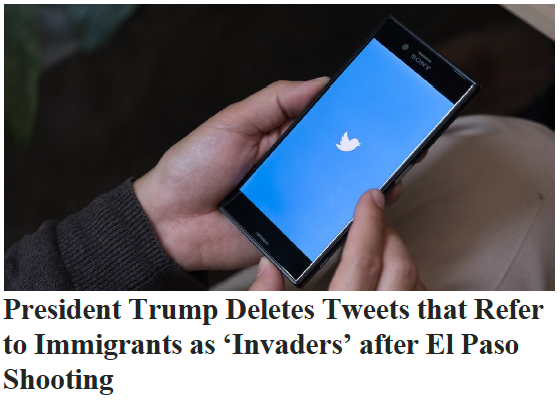

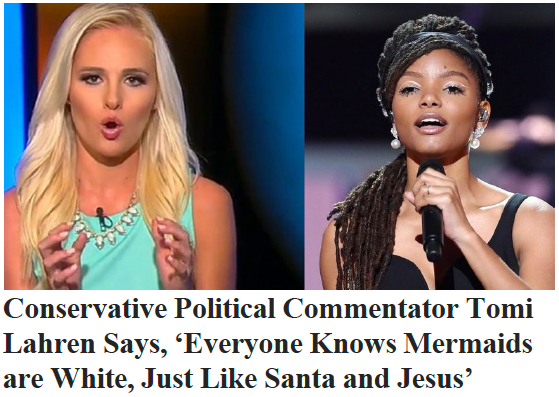
**

Democrat: *M* = 2.73, *SD* = 0.98, *r*(188) = .00, *p* = .988 Democrat: *M* = 2.69, *SD* = 1.05, *r*(188) = .18, *p* = .014

Republican: *M* = 2.29, *SD* = 0.97, *r*(111) = -.04, *p* = .710 Republican: *M* = 2.08, *SD* = 0.97, *r*(111) = -.19, *p* = .041

Other: *M* = 2.56, *SD* = 0.97, *r*(98) = -.02, *p* = .851 Other: *M* = 2.27, *SD* = 0.86, *r*(98) = .04, *p* = .735

**Headline 13: False, pro-conservative Headline 14: False, pro-conservative**

**
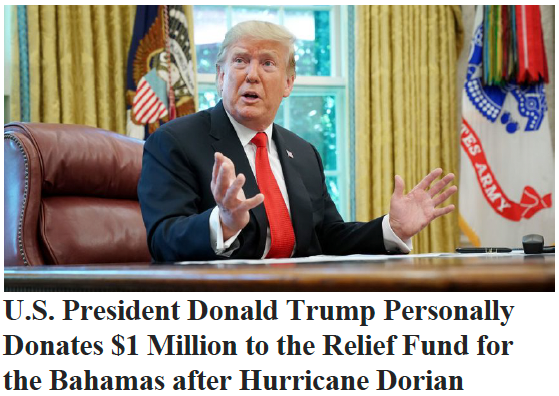

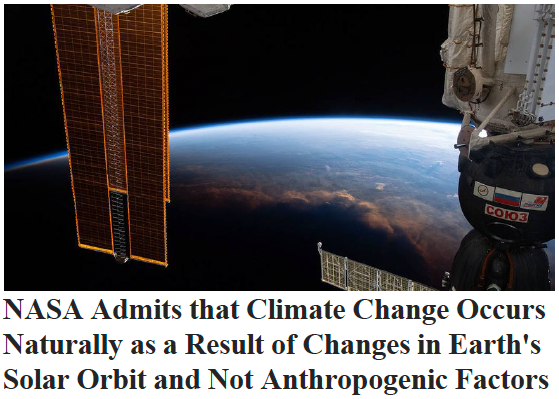
**

Democrat: *M* = 1.93, *SD* = 0.95, *r*(188) = -.17, *p* = .017 Democrat: *M* = 1.73, *SD* = 0.83, *r*(188) = -.17, *p* = .021

Republican: *M* = 2.94, *SD* = 0.93, *r*(111) = -.15, *p* = .123 Republican: *M* = 2.53, *SD* = 1.00, *r*(110) = -.18, *p* = .060

Other: *M* = 2.43, *SD* = 0.95, *r*(98) = -.10, *p* = .346 Other: *M* = 2.25, *SD* = 0.81, *r*(98) = -.14, *p* = .157

**Headline 15: False, pro-conservative Headline 16: False, pro-conservative**

**
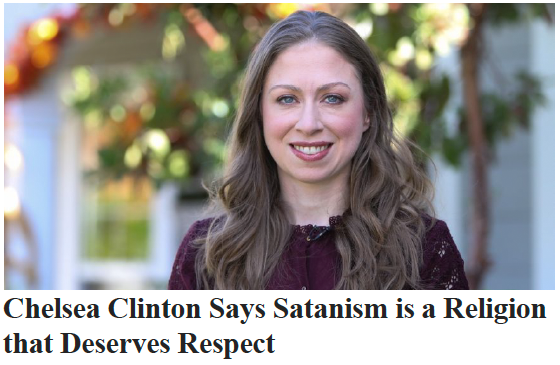

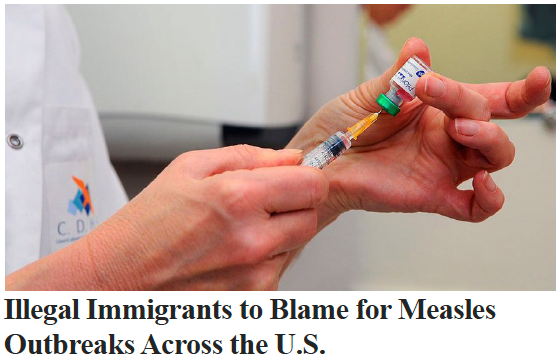
**

Democrat: *M* = 1.69, *SD* = 0.83, *r*(188) = .-.04, *p* = .603 Democrat: *M* = 1.70, *SD* = 0.86, *r*(188) = -.20, *p* = .005

Republican: *M* = 2.27, *SD* = 1.10, *r*(111) = -.06, *p* = .509 Republican: *M* = 2.59, *SD* = 0.94, *r*(111) = -.07, *p* = .441

Other: *M* = 1.80, *SD* = 0.80, *r*(98) = -.13, *p* = .202 Other: *M* = 2.10, *SD* =0.87 , *r*(98) = -.03, *p* = .767

**Headline 17: True, pro-liberal Headline 18: True, pro-liberal**

**
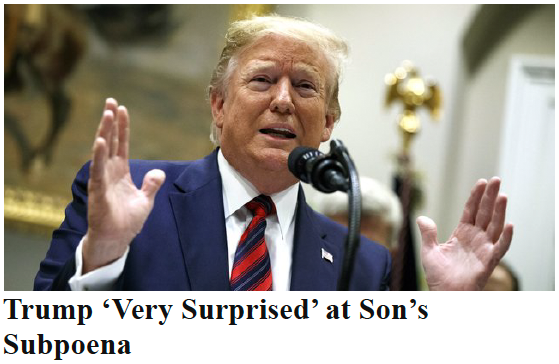

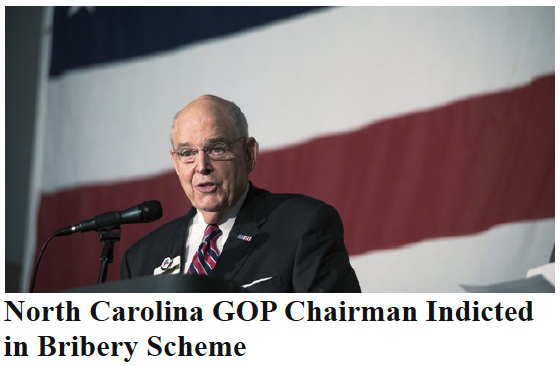
**

Democrat: *M* = 2.99, *SD* = 0.95, *r*(188) = -.04, *p* = .565 Democrat: *M* = 3.08, *SD* = 0.71, *r*(188) = .16, *p* = .033

Republican: *M* = 2.81, *SD* = 0.94, *r*(111) = .12, *p* = .207 Republican: *M* = 2.81, *SD* = 0.85, *r*(111) = -.05, *p* = .620

Other: *M* = 2.76, *SD* = 0.83, *r*(98) = .28, *p* = .004 Other: *M* = 2.84, *SD* = 0.66, *r*(98) = .07, *p* = .480

**Headline 19: True, pro-liberal Headline 20: True, pro-liberal**

**
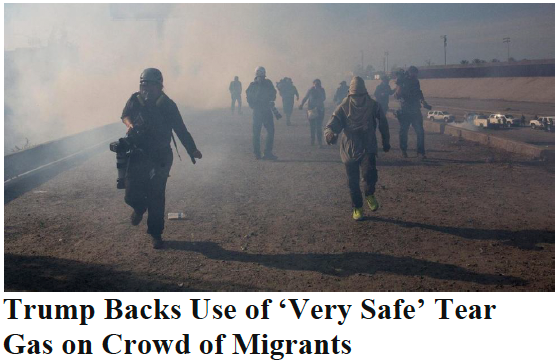

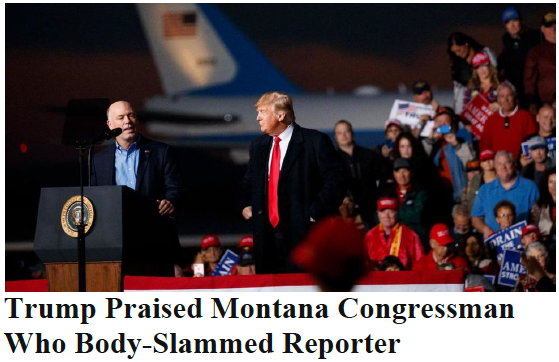
**

Democrat: *M* = 2.82, *SD* = 0.99, *r*(188) = .08, *p* = .296 Democrat: *M* = 3.21, *SD* = 0.92, *r*(187) = .27, *p* < .001

Republican: *M* = 2.51, *SD* = 0.99, *r*(111) = .03, *p* = .750 Republican: *M* = 2.58, *SD* = 0.90, *r*(111) = .03, *p* = .745

Other: *M* = 2.60, *SD* = 0.87, *r*(98) = .02, *p* = .809 Other: *M* = 2.73, *SD* = 0.82, *r*(98) = .13, *p* = .206

**Headline 21: True, pro-conservative Headline 22: True, pro-conservative**

**
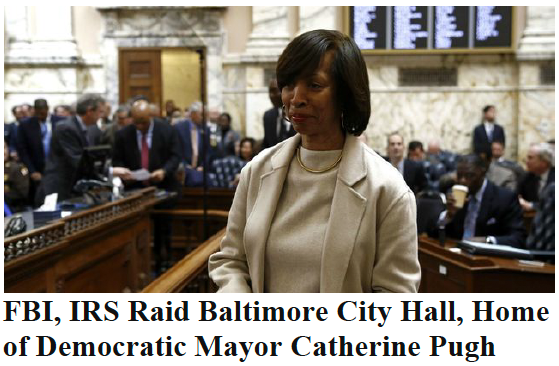

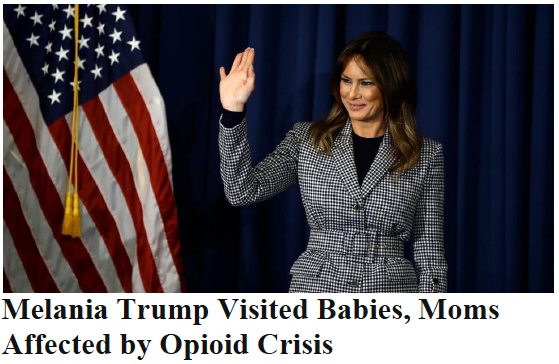
**

Democrat: *M* = 2.60, *SD* = 0.96, *r*(188) = .04, *p* = .629 Democrat: *M* = 2.91, *SD* = 0.87, *r*(188) = -.02, *p* = .811

Republican: *M* = 2.81, *SD* = 0.91, *r*(111) = .10, *p* = .287 Republican: *M* = 3.35, *SD* = 0.65, *r*(111) = .06, *p* = .515

Other: *M* = 2.59, *SD* = 0.88, *r*(98) = .15, *p* = .127 Other: *M* = 3.05, *SD* = 0.72, *r*(98) = .07, *p* = .507

**Headline 23: True, pro-conservative Headline 24: True, pro-conservative**

**
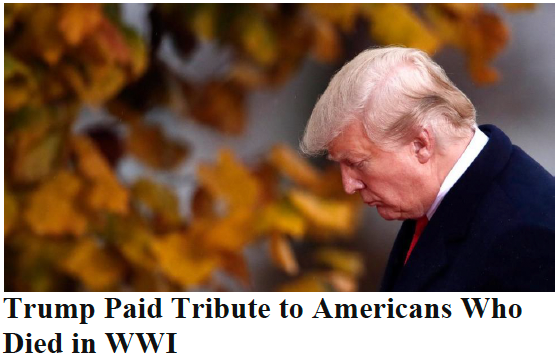

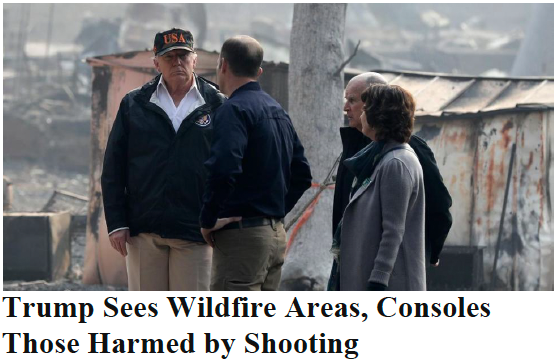
**

Democrat: *M* = 2.87, *SD* = 0.94, *r*(187) = .-.05, *p* = .475 Democrat: *M* = 2.47, *SD* = 0.94, *r*(188) = .06, *p* = .413

Republican: *M* = 3.42, *SD* = 0.79, *r*(111) = -.03, *p* = .760 Republican: *M* = 3.13, *SD* = 0.93, *r*(111) = .20, *p* = .031

Other: *M* = 3.25, *SD* = 0.69, *r*(98) = .02, *p* = .857 Other: *M* = 2.81, *SD* = 0.91, *r*(98) = -.09, *p* = .361

**Headline 25: False, pro-liberal Headline 26: False, pro-liberal**

**
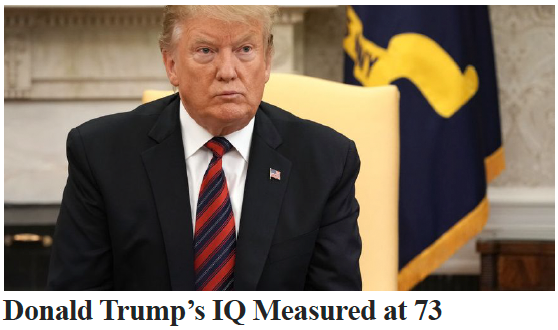

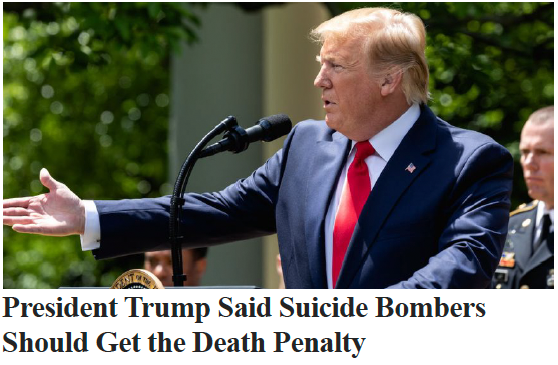
**

Democrat: *M* = 1.94, *SD* = 1.00, *r*(188) = -.21, *p* = .005 Democrat: *M* = 2.92, *SD* = 0.98, *r*(188) = -.06, *p* = .386

Republican: *M* = 1.62, *SD* = 0.86, *r*(111) = -.04, *p* = .676 Republican: *M* = 2.94, *SD* = 1.10, *r*(111) = .11, *p* = .253

Other: *M* = 1.68, *SD* = 0.90, *r*(98) = -.22, *p* = .026 Other: *M* = 2.75, *SD* = 1.00, *r*(98) = -.03, *p* = .773

**Headline 27: False, pro-liberal Headline 28: False, pro-liberal**

**
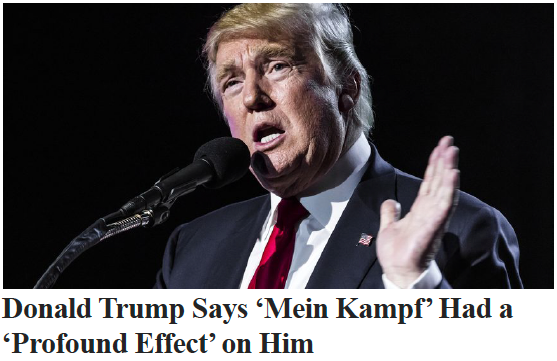

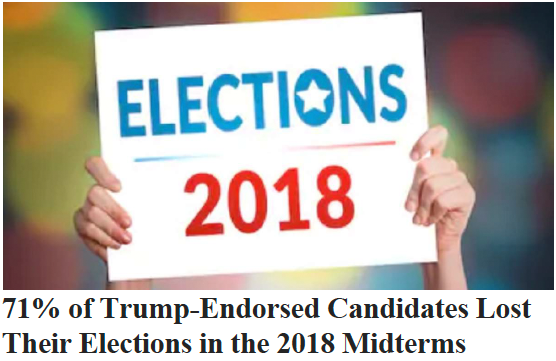
**

Democrat: *M* = 2.41, *SD* = 1.03, *r*(188) = -.06, *p* = .389 Democrat: *M* = 2.71, *SD* = 0.81, *r*(188) = .14, *p* = .049

Republican: *M* = 2.09 *SD* = 0.94, *r*(111) = -.25, *p* = .009 Republican: *M* = 2.18, *SD* = 0.88, *r*(111) = -.09, *p* = .359

Other: *M* = 2.08, *SD* = 0.87, *r*(98) = -.04, *p* = .717 Other: *M* = 2.31, *SD* = 0.75, *r*(98) = -.04, *p* = .683

**Headline 29: False, pro-conservative Headline 30: False, pro-conservative**

**
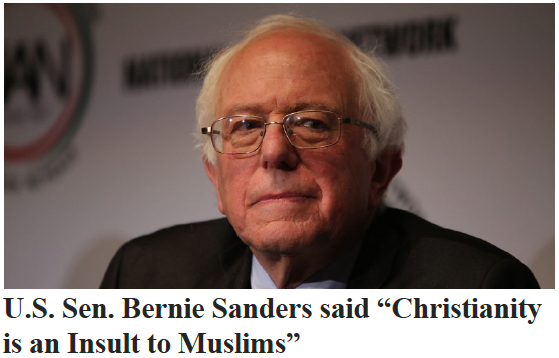

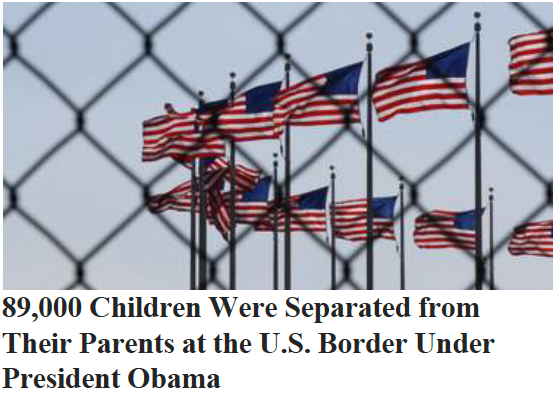
**

Democrat: *M* = 1.63, *SD* = 0.80, *r*(188) = -.17, *p* = .020 Democrat: *M* = 2.45, *SD* = 1.02, *r*(188) = .07, *p* = .367

Republican: *M* = 2.60, *SD* = 0.99, *r*(111) = -.13, *p* = .181 Republican: *M* = 2.95, *SD* = 0.99, *r*(110) = .09, *p* = .351

Other: *M* = 2.04, *SD* = 0.80, *r*(98) = -.10, *p* = .308 Other: *M* = 2.76, *SD* = 0.98, *r*(98) = .09, *p* = .359

**Headline 31: False, pro-conservative Headline 32: False, pro-conservative**


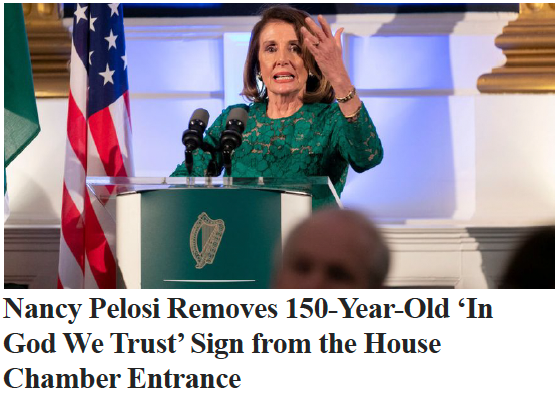

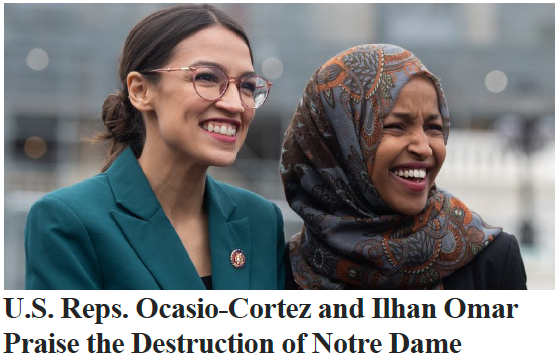


Democrat: *M* = 1.68, *SD* = 0.80, *r*(188) = -.07, *p* = .367 Democrat: *M* = 1.40, *SD* = 0.72, *r*(187) = -.24 *p* = .001

Republican: *M* = 2.51, *SD* = .99, *r*(111) = -.13, *p* = .175 Republican: *M* = 2.20, *SD* = 1.04, *r*(111) = -.20, *p* = .032

Other: *M* = 2.01, *SD* = 0.81, *r*(98) = -.24, *p* = .015 Other: *M* = 1.85, *SD* = 0.83, *r*(98) = -.09, *p* = .393

**Experiment 2 Analysis that includes counterbalanced condition**

To examine the effects of different headlines appearing in different conditions, we conducted an exploratory (not preregistered) analysis. We analyzed which half of headlines participants previously rated as a factor in a four-way mixed model ANOVA (with initial rating task, prior exposure, headline truth, and counterbalanced condition) as independent variables (the first two were repeated measures) and perceived accuracy as the dependent variable. The effects of prior exposure, *F*(1, 399) = 35.46, *p* < .001, η_p_^2^ = .08, and headline truth, *F*(1, 399) = 821.57, *p* < .001, η_p_^2^ = .67, were still significant, and the specific type of initial ratings still did not have a significant effect on perceived accuracy, *F*(1, 399) = 1.41, *p* = .237, η_p_^2^ = .00. Additionally, the counterbalanced condition did not have a significant effect, *F*(1, 399) = 2.00, *p* = .158, η_p_^2^ = .01. Critically, the interaction between prior exposure and initial rating was still significant, *F*(1, 399) = 13.45, *p* < .001, η_p_^2^ = .03. The counterbalanced condition significantly interacted with prior exposure in a two-way interaction and with prior exposure and headline truth in a three-way interaction. Because the three-way interaction occurred, the two-way interaction between counterbalanced condition and prior exposure depended on headline truth. Therefore, we focus on the three-way interaction. We conducted three-way ANOVAs separately for true and false headlines and examined the interactions between counterbalanced condition and prior exposure. With true headlines, this interaction was larger than it was with false headlines, *F*(1, 399) = 48.50, *p* < .001, η_p_^2^ = .11; *F*(1, 399) = 17.62, *p* < .001, η_p_^2^ = .04. The effect of prior exposure was in the predicted direction for only one of the two counterbalanced conditions for true headlines, whereas it was in the predicted direction for both counterbalanced conditions for false headlines. This resulted in a larger two-way interaction between counterbalanced condition and prior exposure with true headlines. Collectively, these results suggest that the effects of prior exposure with true headlines may depend on the headlines.
